# Chronic fluoxetine prevents fear memory generalization and enhances subsequent extinction by remodeling hippocampal dendritic spines and slowing down systems consolidation

**DOI:** 10.1038/s41398-019-0371-3

**Published:** 2019-01-31

**Authors:** Lizeth K. Pedraza, Rodrigo O. Sierra, Marcelo Giachero, Walquiria Nunes-Souza, Fernanda N. Lotz, Lucas de Oliveira Alvares

**Affiliations:** 1 Laboratório de Neurobiologia da Memória, Biosciences Institute, Porto Alegre, 91.501-970 Brazil; 2 Graduate Program in Neuroscience, Institute of Health Sciences, Universidade Federal do Rio Grande do Sul, Porto Alegre, 90.046-900 Brazil; 3 Laboratório de Psicobiologia e Neurocomputação, Biophysics Department, Biosciences Institute, Porto Alegre, 91.501-970 Brazil; 4 Department of Pharmacology, Federal University of Santa Catarina, Florianópolis, Brazil

## Abstract

Fear memory overgeneralization contributes to the genesis and persistence of anxiety disorders and is a central hallmark in the pathophysiology of post-traumatic stress disorder (PTSD). Recent findings suggest that fear generalization is closely related to hippocampal dependency during retrieval. The selective serotonin reuptake inhibitor (SSRI) fluoxetine has been used as a first-line treatment for PTSD; however, how it exerts its therapeutic effect remains a matter of debate. Here, using contextual fear conditioning in rats, we show that chronic fluoxetine treatment prevents fear generalization and enhances subsequent extinction. Moreover, fluoxetine treatment after extinction prevents spontaneous recovery. The mechanism through which fluoxetine affects generalization and extinction seems to be through the postponement of systems consolidation, thereby maintaining hippocampal involvement during retrieval. Such an effect relies on a remodeling of dendritic spines in the hippocampus, as well as the number of mature, mushroom-type spines promoted by fluoxetine treatment. In order to further investigate whether fear generalization is a potential predictor of extinction effectiveness, we categorized a large naive population according to their generalization rate. We found that discriminator rats showed a better extinction profile compared to generalizers, suggesting that the generalization rate predicts extinction effectiveness. Hence, we propose that the therapeutic strategy of choice should take into account the extension of memory generalization, in which therapies based on extinction could induce a better outcome in patients who present less fear overgeneralization. These results open new avenues for the development of interventions that prevent fear generalization by maintaining memory dependency of the hippocampus.

## Introduction

Memory generalization allows animals to extend behavioral repertories to similar situations, contributing to cognitive flexibility. Although generalization can be considered a highly adaptive response, overgeneralization of fear memories contributes to pathological states such as post-traumatic stress disorder (PTSD). In fact, fear overgeneralization is considered a hallmark of the diagnostic criteria for PTSD^1,2^ Accordingly, these patients are unable to restrict fear expression to appropriate predictors, causing fear and avoidance in response to harmless stimuli that are not directly related to trauma^3^.

Behavioral therapies and pharmacological treatments are the most common interventions to attenuate these pathological memories^4^. In exposure extinction-based therapies, traumatic reminders are repeatedly presented in a safe environment, leading to a progressive reduction in fear expression. However, extinction does not erase the original memory but induces new learning that transiently inhibits fear expression. Thus fear memory eventually re-emerges by the passage of time (spontaneous recovery)^5,6^.

Fluoxetine and citalopram are well-known selective serotonin reuptake inhibitor (SSRIs) antidepressant used as a first-line treatment for adult PTSD^7^. In the past decade, the mechanisms underlying the clinical improvement associated with fluoxetine have been thoroughly investigated^8,9^. It has been shown that fluoxetine induces neurogenesis, synaptic plasticity, and dendritic spine remodeling^10–12^. Indeed, the mood-improving effects of fluoxetine depend on dendritic spine remodeling in the hippocampus^12^. An interesting study has shown that 3 weeks of fluoxetine treatment combined with extinction training induces an enduring reduction in the conditioned fear response and prevents spontaneous recovery^13^. Interestingly, this behavioral outcome coincided with increase in synaptic plasticity in amygdala GABAergic neurons that control fear expression^13^. Importantly, other SSRIs such as citalopram have shown the opposite effect, disrupting acquisition and retention of fear extinction^14^.

Surprisingly, few studies have been conducted to explore the effects of SSRIs on fear generalization^15^. Recently, we showed that fear generalization is closely related to hippocampal dependency during retrieval^16,17^; we found that this structure is crucial to orchestrate the reconstruction of detailed memories^16–18^. Environmental factors, such as sequential learning and training intensity, can accelerate hippocampal independency and memory generalization^17,19,20^. Evidence from animal studies shows that retrieval of recent contextual fear memory induces higher hippocampal activation than remote memories^21^. In contrast, several areas of the medial prefrontal cortex (mPFC) are more activated during retrieval of remote memories^22^. As opposed to the hippocampus, these cortical structures seem to be essential for the expression of fear generalized memories^23,24^. Thus the transition from hippocampus dependence to hippocampus independence renders memories into a more schematic, generalized state^25,26^ (for a comprehensive review of these systems consolidation models, see refs. ^27,28^).

Here we tested the effects of chronic treatment with the SSRI fluoxetine and citalopram on contextual fear memory generalization and subsequent performance during fear extinction. Additionally, we explored the close relationship between memory discrimination and fear extinction in naive animals, as a potential predictor of extinction outcome. Our results are discussed in light of the observation that systems consolidation is an important player in the pathophysiology of PTSD and may be a novel approach to be considered for pharmacological and behavioral treatments of fear-related disorders.

## Materials and Methods

### Subjects

Naive, adult male Wistar rats (270–320 g/3 months) from our breeding colony were used. Animals were housed in plastic cages, 4–5 per cage, under a 12 h light/dark cycle at a constant temperature of 24 °C, with water and food ad libitum. Sample size for each group (*n* = 8–15) was estimated based on previous studies of our laboratory^16,17,19,20^. Animals were randomly assigned to treatment groups. All experiments were conducted in accordance with local and national guidelines for animal care (Federal Law no 11.794/ 2008), and the project was approved by the Ethics Committee of the Federal University of Rio Grande do Sul, Brazil.

### Stereotaxic surgery and cannulae placement

Rats were anesthetized with intraperitoneally (i.p.) ketamine/xylazine (75 and 10 mg/kg, respectively) and bilaterally implanted with 22-gauge guide cannulae aimed at dorsal hippocampus (AP −4.0 mm (from bregma), LL ±3.0 mm, DV −1.6 mm) positioned 1.0 mm above of each structure^29^. Following a 1-week recovery from surgery, animals were submitted to the behavioral procedures.

### Drugs

Fluoxetine hydrochloride and Citalopram hydrobromide (Sigma-Aldrich) were dissolved in 0.9% sterile saline. Both drugs were administered in a dose of 10 mg/kg/ml based on previous studies^14,30,31^ and injected i.p. Chronic treatment consisted of daily administration during 21 days after fear conditioning or extinction protocols depending on the experiment performed.

Muscimol (1 µg/µl; Sigma Aldrich) was dissolved in phosphate-buffered saline (PBS) and bilaterally infused (0.5 µl/side) into dorsal hippocampus 15 min before memory retrieval.

### Intracerebral infusion

At the time of infusion, a 27-gauge infusion needle was inserted into the guide cannulae, with its tip protruding 1.0 mm beyond the tip of the cannula and aimed at the dorsal hippocampus. A volume of 0.5 µl was bilaterally infused at a slow rate (20 µl/h), and the needle was removed only after waiting for an additional 30 s.

### Behavioral procedure

#### Contextual fear conditioning (CFC)

The conditioning chamber consisted of an illuminated Plexiglas box, 25 × 25 cm^2^ with a metallic grid floor. During training, rats were placed in the chamber for 3 min, received 4 footshocks (0.7 mA/2 s) separated by a 30 s interval; 30 s after the last shock, they were returned to their homecages.

#### Context apparatus

Conditioning context consisted of an illuminated Plexiglas box of 25 × 25 cm^2^, grid floor of parallel 0.1 cm caliber stainless steel bars spaced 1.0 cm apart, and fan background noise. The novel context was a rectangular box 2/3 the size of the conditioning context, smooth floor, vanilla essence, and without fan background noise.

#### Fear generalization test

Animals were tested for 4 min both in the novel context (Novel) and in the conditioning context (Ctx) without footshocks on days 22 and 23 after training, respectively.

#### Fear extinction session

Subjects were re-exposed to the training context on day 24 without footshocks for 30 min to induce memory extinction.

#### Fear extinction and spontaneous recovery test

Animals were tested for 4 min in the conditioning context (Ctx) 24 h and 22 days after the extinction session in order to evaluate early retention and spontaneous recovery response, respectively.

#### Open field (OF)

The OF chamber consisted of a 50 cm height, 60 × 40 cm^2^ plywood box and a linoleum floor divided into 12 equal rectangles or “sectors.” In addition, floor was divided into two squares, which allowed the definition of central and peripheral areas. The behavior was recorded by video tracking and processed offline. During the 5-min test session, crossings between sectors (locomotor activity) and the time spent in the periphery and center of the apparatus were measured.

### Behavioral scoring

Freezing behavior was registered in real time by an experienced observer who was blind to the treatments. Freezing was defined as the absence of all movements, except those related to breathing. In the OF test, the number of crossings was considered a measure of motor performance, while the time spent in the center or periphery of the field was considered anxiolytic or anxiogenic responses induced by the treatment, respectively.

### Structural plasticity analysis

Dendritic spine visualization and analysis was performed as previously reported by other researchers^32–34^. Concisely, under deep anesthesia (chloral hydrate, 400 mg/kg i.p.), animals were transcardially perfused, first by ice-cold PBS (0.1 M, pH 7.4) and then fixed using ice-cold 4% paraformaldehyde (PFA) (in 0.1 M PBS, pH 7.4). After the brain was removed and postfixed (4% PFA, 24 h, 4 °C), coronal sections (200-µm thick) containing the dorsal hippocampus were obtained with a vibratome and collected in 0.1% PBS. The CA1 dorsal hippocampus was stained with small droplets (<10 µm) of a saturated solution of the lipophilic dye 1,1′-dioctadecyl-3,3,3′,3′-tetramethyl indocarbocyanine perchlorate (Invitrogen; Carlsbad, CA) in fish oil^35^ by microinjection via a patch pipette and positive pressure application^34^. Using a Leica DMI6000 B laser scanning confocal microscope with a ×100× oil immersion from the Laboratório Central de Microscopia Eletrônica, Florianópolis, Brazil, stacks of labeled dendritic segments were collected. The images were deconvolved using LAS AF Lite software (Leica Microsystems, Wetzlar, Germany). A theoretical point spread function was used.

The dendritic spine analysis was achieved manually using the ImageJ software. Dendritic protrusions <3 µm in length and contacting with the parent dendrite were employed for the analysis^33,36,37^. Special consideration was taken to select a single dendritic segment, presumably from different neurons but from CA1 stratum radiatum, in light of the high density of labeled dendrites. Thus, from the *z*-section projection, both the total number and the number of each particular type of dendritic spine normalized to 10 µm of the dendritic segment length was counted with certainty that each spine was counted only once.

Spine types were classified as previously^32,38,39^: type I or “stubby”-shaped dendritic spines, type II or “mushroom”-shaped dendritic spines, and type III or “thin”-shaped dendritic spines. Different measurements were taken for each dendritic protrusion in order to classify them, in brief: the length (dimension from the base at the dendrite to the tip of its head, *L*), the diameter of the neck (measured as the maximum neck diameter, dn), and the diameter of the head (measured as the maximum head diameter, dh)^38^. Thus individual spines were classified into category based on the specific ratios L/dn and dh/dn^32–34,38^.

### Histology for cannulae placement

The position of the cannulae was verified at the end of the experiments. The brains were removed and immersed in a fixation solution of 30% sucrose and 4% PFA. Brains were then frozen and sliced (50-μm coronal sections) using a cryostat. Sections were stained with cresyl violet and subsequently examined to verify the location of the cannulae (Fig. 2a). Statistical analysis considered only animals with correct cannulae placements.

### Statistical analysis

After checking for normality (Kolmogorov–Smirnov (KS) test) and homoscedasticity (Levene test), each relevant phase of the experiment (generalization, extinction and test (extinction test and spontaneous recovery) was analyzed by two-way repeated-measures analysis of variance (ANOVA) with Student–Newman–Keuls (SNK) post hoc test. OF result were analyzed using independent Student's *t* test. Significance was set at *p* < 0.05. Simple linear regression was used to evaluate the relationship between discrimination index and freezing behavior during the extinction test. Cumulative distribution probabilities for total number, mushroom, stubby, and thin dendritic spines per 10 mm of dendritic segment were compared by KS test. Data were also expressed as median (quartile) and compared by Mann*-*Whitney *U* test. *p* < 0.05 was considered statistically significant. For behavioral profile classification, we used the unsupervised learning algorithm expectation-maximization (EM) to divide naive animals between generalizers and discriminators using as input the discrimination index between conditioning and neural context [training ctx/(training ctx + novel ctx)]. This method was used based on previous studies evaluating fear memory discrimination-based populations^40,41^.

## Results

### Chronic fluoxetine prevents fear memory generalization and enhances subsequent extinction

**Fig. 1:**
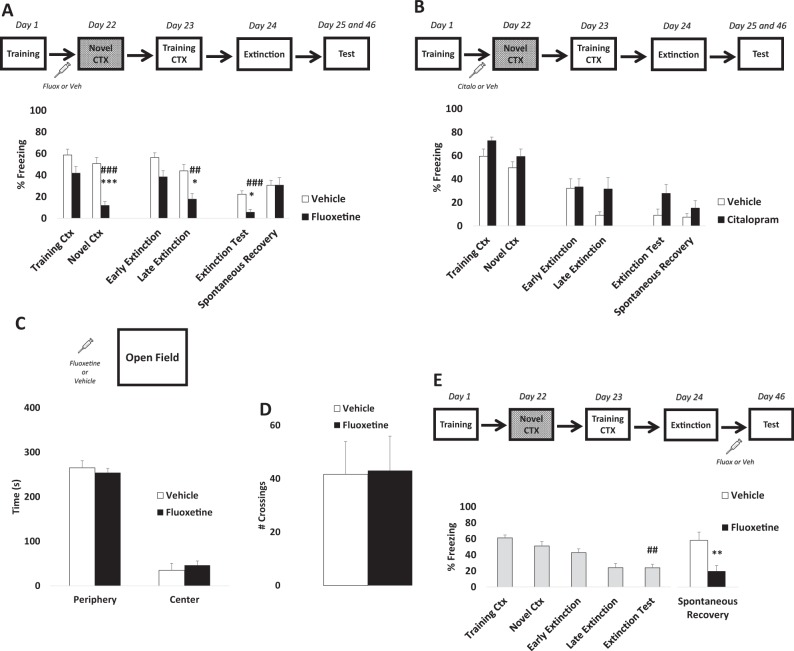
Chronic fluoxetine prevents fear generalization and enhances subsequent fear extinction. The graphs show percentage of freezing time expressed as mean ± SEM, and experimental design is shown at the top of each panel. **a** Animals treated with fluoxetine are able to discriminate between novel and threat context compared to the control group. The same animals showed a better extinction profile (*Differences between groups, ^#^Differences within group. ***p < 0.001, *p < 0.05, ^###^*p* < 0.001, ^##^*p* < 0.01, repeated-measures analysis of variance (ANOVA) followed by Student–Newman–Keuls post hoc test—Vehicle *n* = 13, Fluoxetine *n* = 13). **b** No differences were detected after chronic citalopram administration (ANOVA for repeated measures all factor *p* > 0.05, Vehicle *n* = 9, Citalopram *n* = 10). In addition, fluoxetine effects were not associated with **c** anxiety-like time effects (time spent in the periphery vs. center of the open field (*t* (8) = −0.602; *p* = 0.563, independent *t* test—Vehicle *n* = 5, Fluoxetine *n* = 5)) or **d** locomotion (number of crossings (*t* (8) = −0.078; *p* = 0.939, independent *t* test)). **e** Chronic fluoxetine was able to prevent fear recovery 21 days after the extinction protocol suggesting two potential pharmacological window to attenuate contextual fear memories (*Differences between groups, ^#^Differences within group, **p < 0.01, ^##^*p* < 0.01, independent *t*-test—Vehicle *n* = 12, Fluoxetine *n* = 12)

First, in order to assess the effects of fluoxetine on fear generalization and subsequent extinction, animals were trained in CFC and received daily administration of fluoxetine or its vehicle i.p. for 21 days after training. Twenty-four hours after the last administration, animals were exposed first to a novel context (Novel Ctx) and, on the following day, to the training context (Training Ctx). Twenty-four hours later, animals were submitted to a fear extinction procedure in the training context and memory retrieval was evaluated 24 h and 21 days after extinction (spontaneous recovery). Repeated-measures ANOVA revealed that fluoxetine-treated animals expressed contextual discrimination; less freezing was expressed in the novel context compared to the training context (group × context interaction (*F*_1,24_ = 6.006, *p* = 0.021; SNK post hoc *p* < 0.001)). Moreover, fluoxetine-treated animals expressed less freezing in the novel context than vehicle-treated animals (*p* < 0.001). Vehicle-treated animals were unable to discriminate between the contexts, expressing fear generalization (training vs. novel context *p* > 0.05; Fig. 1a).

Fluoxetine-treated animals expressed a better extinction profile; less freezing was detected during the last 5 min of fear extinction (late extinction) compared to the control group (group × time interaction (*F*_1,24_ = 0.664, *p* = 0.422; SNK post hoc *p* < 0.05)). Additionally, fluoxetine enhanced fear extinction during the test performed 24 h after extinction but did not prevent spontaneous recovery (group × time interaction (*F*_1,24_ = 6.573, *p* = 0.017; SNK post hoc *p* < 0.05). Interestingly, the chronic administration of citalopram, another SSRI, did not prevent fear generalization or alter fear expression (ANOVA for repeated measures all factor *p* > 0.05; Fig. 1b).

These results suggest that chronic fluoxetine (but not citalopram) is able to prevent fear generalization and enhance subsequent extinction. Anxiety-like (time spent in the periphery or center of the OF between groups (*t* (8) = −0.602; *p* = 0.563, independent *t* test); Fig. 1c) or locomotion (number of crossings (*t* (8) = −0.078; *p* = 0.939, independent *t* test)) effects were not induced by the treatment (Fig. 1d).

Although fluoxetine enhanced fear extinction, it did not prevent spontaneous recovery. Prevention of fear relapse after extinction is considered a major challenge for pharmacological and exposure-based therapies^42^. Finally, to verify whether fluoxetine treatment after the extinction training would be able to postpone fear re-emergence, we administered fluoxetine after extinction for 21 days until the spontaneous recovery test. Repeated-measures ANOVA revealed that animals were not able to discriminate between the training and novel contexts. Moreover, a significant reduction in freezing levels was detected between early and late extinction and was maintained during the extinction test (time factor (*F*_4,92_ = 17.670, *p* < 0.001; SNK post hoc *p* > 0.05); Fig. 1e).

After that, animals were randomly assigned to two groups and treated with fluoxetine or vehicle. Our results showed that chronic fluoxetine is able to prevent spontaneous recovery (*t* (22) = 3.055, *p* = 0.005; independent *t* test). This result suggests a dual window of opportunity for pharmacological intervention with fluoxetine to enhance extinction, based on decreasing fear generalization (Fig. 1a) or preventing spontaneous recovery (Fig. 1e).

### Chronic fluoxetine maintains hippocampal dependency during retrieval

Previous studies demonstrate that memory precision involves hippocampal processing during retrieval^17,18^, whereas fear generalization has been associated with an increase in mPFC activity^23,24^ but a decrease in the hippocampus^17,18^. These results are consistent with the systems memory consolidation hypothesis on progressive trace reorganization over time between hippocampal and cortical structures^21,43,44^. Since chronic fluoxetine induces memory precision, we reason that this treatment could promote hippocampal dependency as well. To address this issue, animals were treated with fluoxetine and infused intrahipocampally 15 min before the test in the training context with vehicle or muscimol, a selective agonist for GABA-A receptors able to suppress transiently neural activity. Four hours after the test, a drug-free re-test was performed in the same context. If fluoxetine slows down systems consolidation (keeping hippocampal dependency), then we would expect a strong effect in the test under muscimol.

**Fig. 2:**
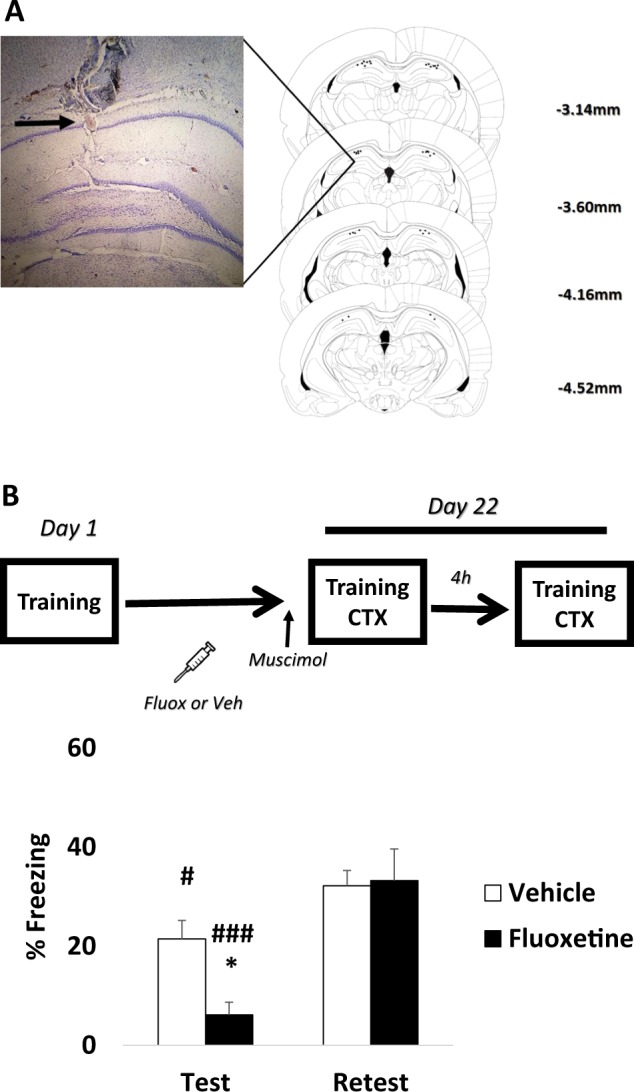
Chronic fluoxetine increases hippocampal involvement during retrieval. The graphs show percentage of freezing time expressed as mean ± SEM, and experimental design is shown at the top of each panel. **a** Examples of Nissl-stained sections and summary of microinjection cannula placements in the dorsal hippocampus. The numbers on the right side of the individual sections indicate the distances from bregma. **b** Although fluoxetine- and vehicle-treated animals were sensitive to intrahippocampal muscimol infusion (test vs. re-test comparison), fluoxetine-treated animals expressed a more robust impairment during retrieval (between-group comparison during test). No differences were detected during the re-test (*Differences between groups, ^#^Differences within group. *p < 0.05, ^###^*p* < 0.001, ^#^*p* < 0.05, repeated-measures analysis of variance followed by Student–Newman–Keuls post hoc test—Vehicle *n* = 11, Fluoxetine *n* = 8)

Repeated-measures ANOVA revealed that fluoxetine-treated animals were more sensitive to intrahippocampal muscimol than the control group during the test (group × time (test vs. re-test) interaction (*F*_1,17_ = 5.371, *p* = 0.033; SNK post hoc *p* < 0.05); Fig. 2b). Thus fear generalization prevented by chronic fluoxetine seems to be strongly associated with hippocampal involvement during retrieval.

### Chronic fluoxetine induces hippocampal structural rearrangement

**Fig. 3:**
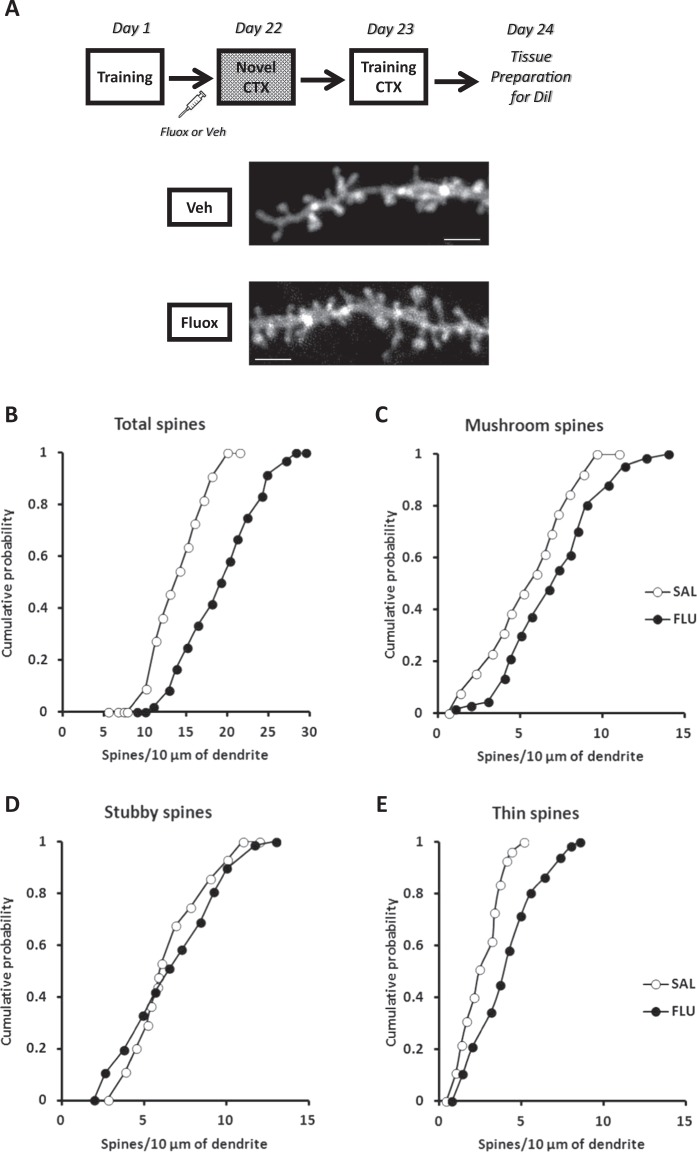
Chronic fluoxetine induces hippocampal structural rearrangement. The graphs show cumulative probability of the spine density. Experimental design is shown at the top of the panel. **a** Representative examples of apical dendritic segments of CA1 dorsal hippocampal pyramidal neurons (stratum radiatum) selected for quantitative analysis of dendritic spines from animals of each experimental group. Bar scale: 2 mm. Cumulative frequency of total (**b**), mushroom (**c**), stubby (**d**), and thin (**e**) dendritic spine (Kolmogorov–Smirnov test—Vehicle *n* = 55 segments, Fluoxetine *n* = 67 segments; 3 rats per group)

Recent evidence suggests that systems consolidation involves an increase in dendritic spine density in the mPFC over time^45^ together with a reduction in dendritic spines in the hippocampus^46^. We hypothesize that hippocampal dependency during retrieval is closely related to dendritic spine density and morphology. Animals treated with chronic fluoxetine or vehicle were perfused, and the brains removed for dendritic spine analysis in the dorsal hippocampus. Spine counts were performed on a total of 122 dendritic segments as follows: control (*n* = 55 segments, 1160.50 µm of total dendritic length analyzed, 3 rats), fluoxetine (67 segments, 1234.51 µm, 3 rats) (Fig. 3a).

Analysis of the cumulative probability distributions for the total density of dendritic spines reflected a significant rightward shift, toward higher numbers of dendritic spines in fluoxetine-treated animals compared to the control group (*p* < 0.05; KS test; Fig. 3b). Moreover, the fluoxetine group also showed a higher median (quartiles, total density/10 µm), 19.0 [13.3–21.4], with respect to the control group, 14.5 [12.1–16.4] (Mann–Whitney *U* test = 1162, *p* < 0.001). Similar to total dendritic spines, a significant rightward shift toward higher numbers of mushroom dendritic spines in the fluoxetine group compared to the control group was observed (*p* < 0.05; KS test; Fig. 3c). In parallel, a higher median (quartiles, mature spines/10 µm) in the fluoxetine group, 7.01 [4.67–8.85], was observed in comparison to the control group, 5.83 [4.38–7.08] (Mann–Whitney *U* test = 1394, *p* < 0.001). The analysis of stubby dendritic spines revealed no significant difference between the experimental groups (*p* > 0.05; KS test; Fig. 3d). There was a comparable median (quartiles, thin spines/10 µm) between the experimental groups, control 5.98 [4.93–7.90], fluoxetine 6.53 [4.58–9.07] (Mann–Whitney *U* test = 1394, *p* < 0.001). For thin dendritic spines, a significant rightward shift toward higher numbers of spines in the fluoxetine group compared to the control group was observed (*p* < 0.05; KS test; Fig. 3e). Similarly, a higher median (quartiles, mature spines/10 µm) in the fluoxetine group, 3.89 [2.45–5.22], was observed in comparison to the control group, 2.45 [1.48–3.41] (Mann–Whitney *U* test = 1043, *p* < 0.001). Our findings suggest that chronic fluoxetine induces a higher density of dendritic spines in the CA1 hippocampal area, indicating a potential mechanism underpinning the maintenance of hippocampal dependency during retrieval and consequently the prevention of memory generalization.

### Discrimination between threat and safe context predicts subsequent fear extinction in naive animals

**Fig. 4:**
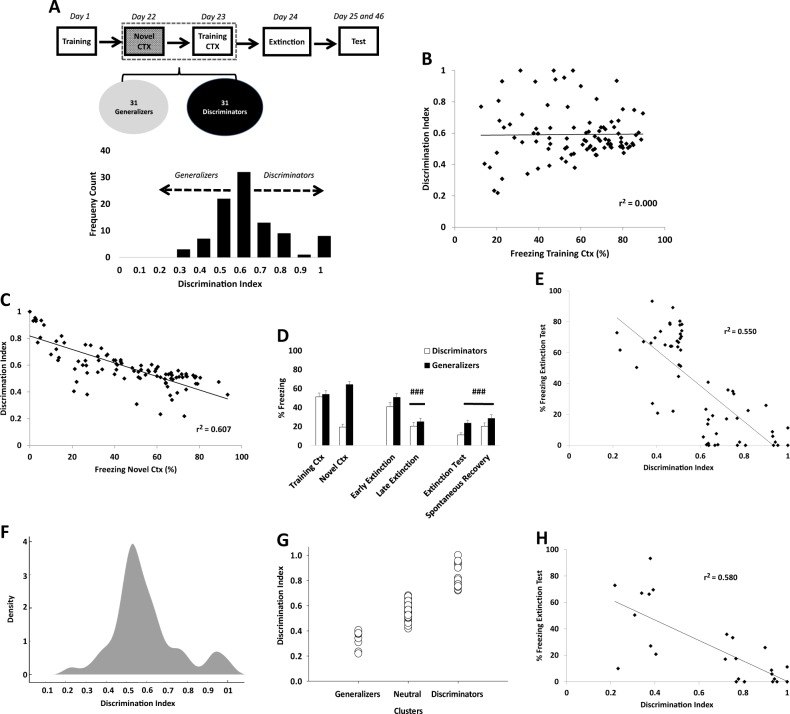
Fear discrimination predicts extinction outcome in naive animals. The graphs show frequency count, percentage of freezing time (mean ± SEM), and linear regression between percentage of freezing time and discrimination index. Experimental design is shown at the top of the panel. **a** The means of higher discrimination were used as a cut-off value to divide total population (*n* = 95) between discriminators (x̄ < 0.51) (*n* = 31) and generalizers (x̄ > 0.61) (*n* = 31). **b** Regression analysis with the total naive population (*n* = 95) revealed no relationship between freezing in the training context and discrimination index (slope did not differ from zero (*F*_1.93_ = 0.013, *p* = 0.909, *r*^2^ = 0.000). However, **c** the same analysis showed a strong relationship between novel context and the discrimination index (slope different from zero, *F*_1,93_ = 144.084, *p* < 0.001, *r*^2^ = 0.607). **d** Animals classified as discriminators expressed less freezing during extinction and spontaneous recovery test compared to generalizers (^#^Differences within group. ^###^*p* < 0.001, repeated-measures analysis of variance followed by Student–Newman–Keuls post hoc test—Discriminators *n* = 31, Generalizers *n* = 31). **e** Linear regression analysis using only discriminators and generalizer populations (*n* = 62) showed a strong relationship between discrimination index and freezing behavior during the extinction test (slope differed from zero, *p* < 0.001, *r*^2^ = 0.550). **f** Gaussian mixture model showing the distribution of the discrimination index. **g** Expectation-maximization (EM) algorithm classified the total population in 3 potential profiles (cluster chosen a priori based on Fig. 5a): generalizers *n* = 9 (9%), neutral *n* = 68 (72%), and discriminators *n* = 18 (19%). **h** Linear regression analysis using only discriminators and generalizer populations (*n* = 27) after EM analysis showed the same strong relationship of Fig. 5e between discrimination index and freezing behavior during the extinction test (slope differed from zero, *p* < 0.001, *r*^2^ = 0.580)

Our first experiment showed that chronic fluoxetine administration prevents generalization and enhances fear extinction. These results suggest that discrimination between the training and the novel context can be considered a potential predictor of the extinction outcome, in which memory generalization would impair fear extinction. To address this possibility, a large number of animals (*n* = 95) were trained and tested in the same conditions as the first experiment, without any pharmacological treatment. First, we calculated a discrimination index [training ctx/(training ctx + novel ctx)] for each animal in order to determine a frequency distribution (Fig. 4a). This analysis allowed us to identify that the vast majority of animals have a discrimination ratio between x̄ = 0.51 and 0.61 (*n* = 33). This range was used as a cut-off value to divide the total population into two specific profile: generalizers (x̄ < 0.51) and discriminators (x̄ > 0.61).

A regression analysis conducted on the freezing levels in the training context and the discrimination index revealed that there was no relationship between these factors, since the slope did not differ from zero (*F*_1.93_ = 0.013, *p* = 0.909, *r*^2^ = 0.000; Fig. 4b). However, a strong relationship was found between the novel context and the discrimination index (slope different from zero; *F*_1,93_ = 144.084, *p* < 0.001, *r*^2^ = 0.607; Fig. 4c). This result suggests that the freezing expressed in the novel context drives fear discrimination.

After analyzing the behavioral profile of the total population, 31 animals were classified as discriminators and 31 as generalizers. As expected, repeated-measures ANOVA revealed higher discrimination and generalization rates (group × context interaction (*F*_1,60_ = 86.420, *p* < 0.001; SNK post hoc *p* < 0.001). However, both groups expressed the same freezing levels in the training context (Fig. 4d).

Both groups were able to extinguish the fear response (time factor (early vs. late) (*F*_1,60_ = 62.435, *p* < 0.001). However, during the extinction and spontaneous recovery test, discriminators expressed less freezing levels than generalizers (group factor (test vs. spontaneous recovery) (*F*_1,60_ = 4.278, *p* < 0.042). This result suggests that discrimination between the training and the novel context is closely associated with a better extinction profile.

Surprisingly, regression analysis revealed a strong relationship between the discrimination index and freezing behavior during the extinction test (slope different from zero; *F*_1,60_ = 73.389, *p* < 0.001, *r*^2^ = 0.550; Fig. 4e).

To further investigate whether these results could be influenced by the data selection method to generate discriminators and generalizers groups, we employed the unsupervised learning algorithm EM in the total population (*n* = 95). This algorithm estimates the maximum likelihood parameters from Gaussian mixture model^40,41^ (Fig. 4f). Number of clusters were chosen a priori based on our previous result (Fig. 4a). This analysis reduced the number of generalizer assigned animals to 9 (9%) and 18 (19%) for discriminators (Fig. 4g). However, even after decreasing the number of animals per group we found the same strong correlation between the discrimination index and freezing behavior during the extinction test (simple linear regression, slope different from zero; F_1,25 _= 34.581, *p* < 0.001, *r*^2^ = 0.580; Fig. 4h).

Taken together, these results demonstrate, for the first time, that memory discrimination can be considered as a potential predictor of the extinction outcome. Our results were supported by two different methods of behavioral categorization. We conclude that better fear extinction profile can be reached as a natural consequence of better discrimination between threat and safe.

## Discussion

The current study showed that chronic fluoxetine administration after training, but not citalopram, prevents fear generalization and enhances subsequent fear extinction (Fig. 1a–b). Although fluoxetine after training did not prevent spontaneous recovery, a persistent fear reduction was reached when fluoxetine was chronically administered after extinction (Fig. 1e). Interestingly, fluoxetine increases hippocampal dependency, indicating that the treatment is able to delay the systems consolidation process (Fig. 2b). This result was accompanied by a higher density of dendritic spines in the CA1 region in animals treated with fluoxetine (Fig. 3a–e). Finally, in a large naive population, animals categorized as good discriminators showed a better extinction profile (similar to fluoxetine-treated animals), indicating that fear discrimination predicts extinction outcome (Fig. 4b–h).

Antidepressant drugs have been used for decades as the first-line pharmacological treatment for PTSD^47^. However, there are several conflicting reports on their efficacy with and without exposure therapies^48,49^. Our findings show that chronic fluoxetine, but not citalopram, prevents fear generalization and enhances fear extinction. This differential effect between antidepressant drugs of the same class in not entirely new. In fact, Burghardt et al.^14^ have shown that chronic citalopram administration impaired the acquisition of fear extinction, an effect closely related to the inhibition of NR2B-NMDA downregulation in lateral and basal nuclei of the amygdala. These results contrast with the fear reduction enhancement promoted by chronic administration of fluoxetine in similar experimental settings^13^. Moreover, the antidepressant escitalopram is able to attenuate mood and anxiety dysfunction in a rodent PTSD model; however, it is insufficient to revert the impaired fear extinction^50^. Taking together, SSRIs seem to modulate differentially fear-related responses, an observation currently validated in clinical population^49^. Importantly, fluoxetine changes the quality, rather than the strength, of fear memories, since no difference was detected in freezing behavior in the training context, but there was a difference in the novel context.

Prevention of spontaneous recovery after extinction is one of the main challenges of behavioral and pharmacological therapies^42^. In our protocol, post-conditioning chronic fluoxetine was able to enhance extinction of contextual fear memories, as previously reported^13,51^. Moreover, a persistent fear reduction was achieved when chronic fluoxetine was administered after extinction training. Altogether, these results indicate a dual window of opportunity to avoid fear memory: (i) by enhancing fear extinction when fluoxetine is administered after conditioning, via slowing of fear generalization, and (ii) by preventing fear recovery after extinction.

A previous study has shown that fear generalization can be prevented by pharmacological treatments targeting glucocorticoid (Metyrapone) and noradrenergic (Propranolol) systems during training^17^. Interestingly, in that study, fear discrimination was closely associated with hippocampal involvement during retrieval. Here chronic fluoxetine administration was able to increase hippocampal dependency and prevent fear generalization, suggesting that fluoxetine delays the systems consolidation rate. The transition from hippocampus dependence to hippocampus independence is thought to render memories into a more schematic, generalized state^25,26^. Additionally, remote memories submitted to this reorganization process seem to be less susceptible to modification or to update^52,53^ and are largely influenced by fear incubation^54^. We suggest that manipulating the systems consolidation rate (i.e., by slowing it down) allows the expansion of the therapeutic window for treating fear-related disorders. This means that preserving hippocampal involvement during retrieval and fear discrimination could be a potential clinical approach to enhance subsequent fear extinction.

**Fig. 5:**
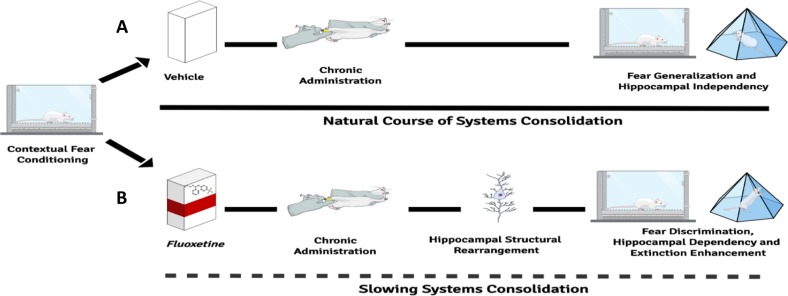
Summary of our findings. **a** Fear learning is able to trigger structural reorganization over time between hippocampal and cortical structures. As a consequence of this systemic process, detailed fear memories are transformed into a more schematic state, inducing fear generalization (the inability to restrict the fear expression to appropriate predictors, causing fear and avoidance in response to harmless stimuli such as the novel context that is not directly associated with trauma). **b** Chronic fluoxetine treatment after contextual fear conditioning prevents fear generalization by maintaining fear memory dependency of the hippocampus. The mechanism underlying such process seems to be the remodeling of dendritic spines densities, especially the mushroom type in the CA1 region. Our findings suggest that manipulating the natural course of systems consolidation can be considered as a potential clinic strategy to treat fear-related disorders through its extinction facilitation

The hippocampal dependency induced by chronic fluoxetine was accompanied by a remarkable structural rearrangement in the CA1 region. Remodeling of dendritic spines seems to guide memory reorganization between hippocampal and cortical structures^46,55–57^. Recently, it has been shown that an immature engram is formed in the mPFC after training, before the process of systems consolidation occurs over time. This maturation process involves a substantial increase in dendritic spines in the mPFC, a structure previously associated with retrieval of generalized memories^23,24^, and a decrease in the hippocampus^45,46^. In our study, fluoxetine treatment modulates remodeling of dendritic spines in the hippocampus, particularly the mature, mushroom type. We assume that the increase in dendritic spines in the hippocampus is one of the mechanisms underpinning the maintenance of hippocampal dependency over time (by slowing down systems consolidation) thus prevent fear generalization and ultimately facilitates extinction. That is, both systems consolidation and memory generalization are controlled by a switch of hippocampal–cortical spine remodeling susceptible to manipulation, which in turn affects subsequent fear extinction (see Fig. 5 for a summary of the findings).

The hypothesis that fear discrimination predicts the extinction outcome was confirmed in subsequent experiments in a large naive population; these studies showed a strong correlation between the discrimination rate and fear extinction using two different methods of behavioral categorization. Indeed, animals classified as good discriminators expressed a similar extinction profile to fluoxetine-treated animals. This result indicates that basal fear discrimination guides fear reduction during/after extinction. Therefore, interventions that keep memory precise and prevent fear generalization would be a promising therapeutic strategy to enhance fear extinction in PTSD.

Although SSRIs such as fluoxetine have been widely used to treat psychiatric disorders, the mechanisms responsible for the clinical improvements are still a matter of debate^8,9^. This study shed new light on the neurobiological process involved in the therapeutic mechanisms of fluoxetine. In fact, it has been previously shown that the mood-improving effects of fluoxetine rely on hippocampus remodeling via dendritic spine remodeling^12^. In the current study, we confirmed this finding; we observed a spine remodeling induced by chronic fluoxetine treatment and extended those findings by revealing an association between dendritic spine morphology, fear generalization, and the maintenance of hippocampus-dependent memory. However, one remaining question after the experimental setting shown here and in other studies^13,14^ are the molecular and structural difference induced by fluoxetine and citalopram treatment that induce different outcomes in fear conditioning experiments.

In conclusion, we have shown that (i) chronic fluoxetine but not citalopram enhances fear discrimination and subsequent fear extinction; (ii) fluoxetine is effective in preventing fear recovery when administered after extinction training; (iii) fluoxetine slows down systems consolidation; and (iv) fluoxetine increases dendritic spine density in the hippocampus. Finally, we showed, for the first time that (v) individual differences in fear discrimination predict extinction performance. These results offer a new strategy for the treatment of fear-related disorders based on maintaining fear discrimination and hippocampal dependency. Our findings could contribute to explaining variable responses to pharmacological and behavioral interventions.
